# Influence of Hypoxia, Dehydration and Salinity on Survival of *Orthohalarachne* Marine Mite Larvae: Limits to Dispersion

**DOI:** 10.3390/biology15050444

**Published:** 2026-03-09

**Authors:** Lucía Pérez Zippilli, José Emilio Crespo, Juan Pablo Loureiro, Dolores Erviti, Marcela Karina Castelo

**Affiliations:** 1 Laboratorio de Entomología Experimental-Grupo de Investigación en Ecología de los Mares (LEE-GIEM), Instituto de Ecología, Genética y Evolución de Buenos Aires (IEGEBA-CONICET-UBA), Departamento de Ecología, Genética y Evolución, Facultad de Ciencias Exactas y Naturales, Universidad de Buenos Aires, Ciudad Universitaria, Pabellón II, Buenos Aires C1428EGA, Argentina; luciaperezzippilli@gmail.com (L.P.Z.); jose.crespo@conicet.gov.ar (J.E.C.); 2 Fundación Mundo Marino, Av. X 157, San Clemente del Tuyú B7105GDB, PBA, Argentina; juanploureiro@gmail.com (J.P.L.); ervitidolores@gmail.com (D.E.)

**Keywords:** *Acari*, marine mammals, mites, *Otariidae*, parasites

## Abstract

*Orthohalarachne attenuata* and *O. diminuata* mites are parasites of the respiratory system of Pinnipeds. During hosts’ dives, mites must cope with changing conditions of oxygen availability in the nasal cavity. Adults and nymphs live inside the host, but larvae are active and responsible for colonizing new hosts. Hence, larvae are also exposed to environmental conditions with variable temperature and pressure, as well as to dehydration and changes in salinity. Although both species live within the respiratory tract of hosts, adults attach to different sections. Also, larvae have differential thermal tolerances and locomotion capacities. In this study, we show the effect of hypoxia, humidity and salinity on survival of *O. attenuata* and *O. diminuata* mite larvae. We found that both species are highly tolerant to hypoxia and can withstand it for long periods. In turn, both species showed low survival when exposed to direct air. Finally, hyperosmotic solution was highly harmful for *O. attenuata*, but not for *O. diminuata*. Our results show that humidity rather than oxygen availability is a constraint for survival and a limitation for dispersal when searching for new hosts. The present study expands our knowledge of ecophysiology and adaptations to changing conditions experienced during the dispersal of these marine parasite species.

## Simple Summary

The present study examines the responses of *O. attenuata* and *O. diminuata* mite larvae from *A. australis* to low oxygen levels and conditions of humidity and salinity. The objective of this study was to ascertain the impact of environmental factors on survival of mite larvae within the nasal cavity during their development and outside the host during dispersal. We found that both species have high resistance to oxygen deprivation, and that humidity is the primary limiting factor outside the host. Also, both species show different rates of survival under stressful salinity conditions, with *O. attenuata* less tolerant than *O. diminuata*. In conclusion, *O. attenuata* and *O. diminuata* larvae have physiological capabilities to tolerate environmental stresses both inside and outside the host, suggesting ecophysiological adaptations relevant to their parasitic lifestyle.

## 1. Introduction

Marine mites of the family *Halarachnidae* Oudemans, 1906 (*Acari*: *Parasitiformes*: *Mesostigmata*: *Dermanyssoidea*), are obligate endoparasites that infest the respiratory tracts of Pinnipeds [1]. As such, they are exposed to a stable host internal medium. However, mites are also challenged to cope with changing conditions of oxygen availability in the nasal cavity whenever hosts dive. Hosts present a “dive response”: a set of physiological responses that allow prolonged apneas through a significant reduction in blood flow to several organs, bradycardia and peripheral vasoconstriction that saves blood oxygen for central hypoxia-intolerant organs such as the lungs, heart and brain [2]. Thus, whenever hosts dive, the available oxygen is reduced. Oxygen uptake by mite larvae occurs first by diffusion through the cuticle because they do not have a well-developed trachea [3].

Besides being exposed to the changing conditions inside their hosts, larvae can be expelled through sneezing in response to nasal inflammation and congestion of the upper respiratory mucosa of hosts when heavily infested [4,5]. After being expelled, *Halarachindae* larvae must survive environmental conditions like variable temperatures and changes in salinity, as well as dehydration, if they fall in the intertidal zone. Then, actively crawling, larvae must find the nostrils of a new host [4]. Very little information is available on the survival of mites under different stressful conditions of reduced amounts of oxygen, dehydration or salinity (but see [6]).

*Orthohalarachne attenuata* (Banks, 1910) and *O. diminuata* (Doetschman, 1944) are the two known species that infest *Arctocephalus australis* (Zimmermann, 1783) (South American fur seal) and *Otaria flavescens* (Shaw, 1800) (Southern sea lion) (Carnivora: *Otariidae*) in South America [7,8,9,10,11,12,13]. Both mite species can co-occur in the same host, but the final anchorage location of the adult to the host is different. Adults and larvae of *O. attenuata* reside in the turbinates and nasopharyngeal mucosa, whereas *O. diminuata* adults are found in the lungs, larvae are found in the turbinate mucosa, and nymphs are less observable and are thought to migrate along the tract [5].

Adults are almost motionless, and they live all their lives attached to the respiratory mucosa of their hosts. Larvae are the dispersive stage and can exit the host during nose-to-nose contacts or be expelled to the outside, where they must survive environmental conditions to locate and infest a new host [4,5]. If sneezing as a dispersal mechanism is an important way of transmission, larvae would be exposed to a wide range of temperatures besides those experienced within the host. It has been already shown that, despite interspecific differences, larvae of both species can tolerate a wide range of temperatures compatible with this way of transmission [13]. However, it is unknown if larvae can tolerate the changes in available oxygen when hosts dive, or how changes in humidity and salinity influence survival whenever larvae are expelled from their hosts.

In this work, we study the responses of *O. attenuata* and *O. diminuata* from *A. australis* to immersion and the survival of mite larvae when exposed to different conditions of humidity and salinity. In particular, we aim to understand the influence that different environmental conditions can exert on the survival of mite larvae. One of our working hypotheses is that larvae should be able to tolerate periods of hypoxia given that hosts dive frequently for feeding. Then, we hypothesize that there are interspecific differences in tolerance to different conditions of humidity and salinity between larvae of *O. attenuata* and *O. diminuata*. However, both species will show enough tolerance to allow them to locate new hosts.

## 2. Materials and Methods

### 2.1. Ethical Note

No licences or permits were required for this research. All hosts used during the experiments died naturally in Mundo Marino Foundation’s Rescue and Rehabilitation Centre. During experiments, the mite larvae were not harmed. After the experiments, the larvae were kept in saline solution until death.

### 2.2. Collection of Mites

**Figure 1:**
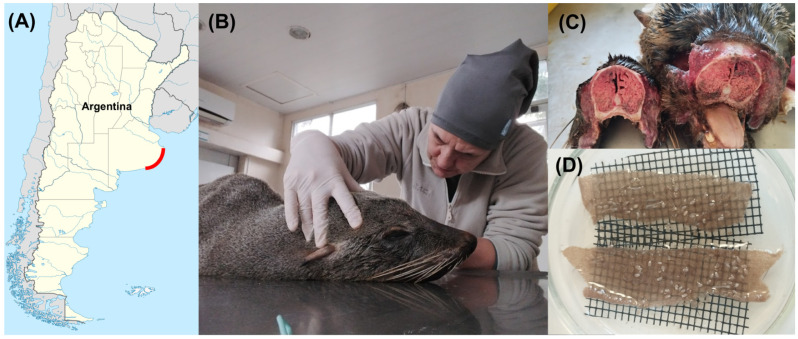
Collection of mites. (**A**) Red line: Buenos Aires Province coast in Argentine Sea, Argentina, where stranded hosts were found. (**B**) Stranded *A. australis* host. (**C**) Turbinates and nasopharyngeal tissues after the necropsy. (**D**) Petri dish with mite larvae of both species.

Stranded *A. australis* hosts were found in localities of the Buenos Aires Province coast, Argentine Sea, Argentina, in August 2025, and taken for recovery to Mundo Marino Foundation’s Rescue and Rehabilitation Centre (Figure 1A). Whenever a host died, it was kept in a fridge at 3 °C until the performance of the necropsy to separate the respiratory system (Figure 1B). After performing the necropsies, the turbinates and nasopharyngeal tissues were separated (Figure 1C). Then, *O. attenuata* and *O. diminuata* larvae were manually collected under a stereomicroscope (Olympus SZ61, Olympus Corporation, Tokio, Japan) using forceps and a paintbrush and placed in glass Petri dishes with 0.9% NaCl saline solution (Tecsolpar^®^, Balcarce, Argentina). Mites were taxonomically identified under a stereomicroscope [5,11,14]. A piece of cloth and fabric were placed at the base of the Petri dish to allow mites to attach (Figure 1D). The saline solution was replaced every day. Mites were kept at a room temperature of 25 ± 1 °C and a 12:12 light/dark cycle until used in the experiments. In total, mites from four different hosts were used (Mundo Marino Foundation ID M9425 and M10025 adult females, M9625 and M9925 juvenile females).

### 2.3. Experimental Design

#### 2.3.1. Response of Mites to Hypoxia and Dehydration

**Figure 2:**
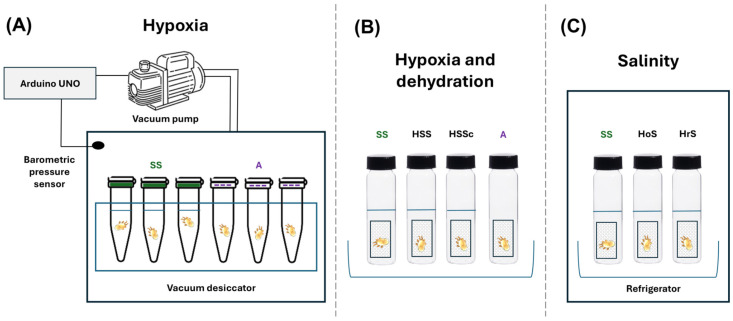
Experimental devices used to study the effects of hypoxia, dehydration and salinity on both species of *Orthohalarachne* mites. Each individual mite was placed either in a tube or in a vial. (**A**) Device to study the effects of hypoxia on survival. A vacuum desiccator was modified to be controlled by an Arduino UNO. A vacuum pump was controlled to generate the different oxygen treatments. Blue line: Liquid level. Dotted line: Mesh fabric. (**B**) Set-up to study the effects of hypoxia and dehydration on survival simultaneously. Vials contained saline solution with different oxygen concentrations. (**C**) Set-up to study the effects of salinity on survival. Vials contained solutions with different concentrations of NaCl. SS: Saline solution, A: Air, HSS: Hypoxic saline solution, HSSc: Hypoxic saline solution control, HoS: Hypoosmotic solution, HrS: Hyperosmotic solution.

In order to determine the ability of *O. attenuata* and *O. diminuata* to endure hypoxia conditions, we set up an experimental device consisting of a modified SB-type acrylic vacuum desiccator (Sanplatec Corporation, Osaka, Japan). This chamber has internal dimensions of 20 cm × 20 cm × 19.5 cm with a lid fitted with a silicone gasket to ensure that the sealed chamber is completely hermetic. The lid also has a valve with a nozzle and a vacuum gauge measuring system. The chamber was modified to be fully controlled by the low-cost and easy-to-use Arduino technology, which has already been used in the laboratory to develop other experimental devices. We used an Arduino UNO with a BMP085 pressure sensor (Bosch Sensortech, Suzhou, China) to take measurements of the barometric pressure. A vacuum pump (Dvr II series, Dosivac, San Martín, Argentina) was connected to the chamber’s valve system through a tube and controlled by the Arduino through a solid-state relay (Dhacel, Villa Lynch, Argentina) (Figure 2).

First, we studied if larvae of *O. attenuata* and *O. diminuata* are capable of surviving hypoxic conditions. To accomplish this, we exposed larvae to a chamber pressure of 100 hPa for 10 min. Larvae were placed in individual 0.5 mL Eppendorf-type tubes that were either filled with saline solution (N = 8 *O. attenuata*, N = 16 *O. diminuata*) or simulated dry conditions (N = 6 *O. attenuata*, N = 11 *O. diminuata*). For the dry-condition assays, we perforated the lids of the tubes, adding a mesh fabric to prevent the mites from escaping. In each trial, tubes were placed in a rack inside the chamber (Figure 2A). After the time elapsed, we evaluated the status of the larvae. A larva was alive if it was turgid and it showed movement of appendages after gentle disturbance with a small paintbrush. If the larva was flabby and it showed no sign of movement, it was considered dead.

Given that larvae had no problem surviving one exposure to hypoxic conditions, we then exposed larvae to increasing times of 60 min (N = 13 *O. attenuata*, N = 12 *O. diminuata*), 300 min (N = 12 *O. attenuata*, N = 13 *O. diminuata*) and 960 min (N = 20 *O. attenuata*, N = 15 *O. diminuata*) at an air pressure of ~1 hPa. We placed larvae in the same tubes as before, but only in the dry-condition tubes (Figure 2A). The reason for only using dry conditions was that with our set-up, we were confident that we were manipulating air pressure. Dissolved oxygen was not directly measured because the pressure sensor measures air pressure. As before, we evaluated the status of larvae after the exposure time elapsed.

Finally, we evaluated the simultaneous effects of hypoxia and dehydration on survival of larvae. To accomplish this, we placed larvae in 2 mL vials with a small piece of plastic mesh netting, in accordance with the treatments listed in Table 1 (Figure 2B). In each vial, except for the air treatment, three drops of normal saline solution (0.9%), enough to cover the larva, were introduced with a pipette. Larvae were evaluated every 24 h for 96 h following the same criterion as before, and their survival was registered. To generate a hypoxic medium, we boiled saline solution for 15 min and placed three drops of this solution with a pipette into the vials. Because the vials were closed, equilibrium with atmospheric air could not be reached. However, because we opened each vial every 24 h to check for survival of larvae, air could perhaps get inside the vials during the small amount of time that the flask was open. We performed another control series with only *O. attenuata* larvae because *O. attenuata* was the only species for which we had enough larvae to test. In this control, we exposed three different groups of larvae to hypoxic saline solution, as before, but groups of larvae were checked either at 24 h (N = 30), 72 h (N = 6) or 168 h (N = 6). Because the vials were opened only at the end of the time of exposure, we are certain that no oxygen exchange occurred until larvae were evaluated.

#### 2.3.2. Response of Mites to Salinity

In order to study the influence of salinity on the survival of *O. attenuata* and *O. diminuata* larvae, three solutions with different levels of salinity were tested (Table 2). As a control, we used 0.9% NaCl saline solution, which is isosmotic to the respiratory mucosa of the host. Then, we exposed larvae to either a solution of tap water (hypoosmotic solution) or salted water (hyperosmotic solution). Salted water was made by adding 35 g/L of NaCl to distilled water. Mite larvae were placed individually inside 2 mL vials with a small piece of plastic mesh netting to which they could attach (Figure 2C). Three drops of each solution were put inside each vial to completely cover the mite’s body. Mites were then kept at a temperature of 7 ± 1 °C. Individuals were assessed for survival every 24 h for 96 h. As before, larvae were considered dead if they exhibited no movement of appendages or if a lack of body turgor was registered after a gentle touch with the paintbrush.

### 2.4. Statistical Analysis

In order to study if larvae are capable of surviving hypoxic and different humidity conditions, a generalised linear model (GLM) was used. The probability of mite larvae surviving was analysed with a GLM with logit link function and binomial distribution. The logit link function ensures fitted values between 0 and 1, and the binomial distribution is typically used for probability data. If the mite larva showed any movement, it was counted as alive with a 1; otherwise, it was assumed dead, and a 0 was registered.

To study the response of larvae to hypoxic conditions (100 hPa), the predictors used were treatment (fixed categorical predictor with two levels, saline solution and dry) and species (fixed categorical predictor with two levels, *O. attenuata* and *O. diminuata*). Then, in order to study the influence of exposure time to hypoxic conditions on survival (1 hPa in air), the model included species as a predictor (fixed categorical predictor with two levels) and time of exposure (fixed continuous predictor). The models included fixed factors and their interaction.

To study the effect of hypoxia and humidity on larvae’s survival, we constructed a Cox Proportional Hazards Model. We evaluated the influence of the medium (with the levels air, hypoxic solution, and saline solution) on the hazard rate. We included the species as a stratification variable. Stratification allowed each species to maintain its own baseline hazard function, ensuring that the model accurately accounted for inherent species–specific differences in survival time. The response variable was the time to death (in h), with the event status (death or censoring) coded in the status variable. When evaluating the assumption of proportional hazards, we found a significant violation for the medium predictor (*p* = 0.047). Hence, as this indicated that the effect of the medium on the risk of mortality was not constant over the observation period, we incorporated a time-dependent covariate for the medium. In this way, we explicitly modelled the interaction between the medium levels and the log of time, allowing the hazard ratio (HR) for each treatment to vary temporally. The concordance of the model was calculated as a measure of the discriminatory power of the model. Given that while checking survival for the hypoxic saline solution, the vial was opened for a short time, and that this could have allowed some air to get inside, we analysed the proportion of live larvae in saline solution, hypoxic saline solution and hypoxic control solution at 72 and 96 h by means of a Fischer’s exact test with Monte Carlo simulations to account for the small number of replicates in some of the groups.

Finally, to analyse the effect of salinity on larvae survival, we constructed a Cox model as before. In this model, the medium (with the levels isosmotic saline solution, hypoosmotic solution and hyperosmotic solution) and the species (with the levels *O. attenuata* and *O. diminuata*) were evaluated. In this case, the model was in accordance with the assumption of proportional hazards. The response variable was the time to death (in h), with the event status (death or censoring) coded in the status variable. The concordance of the model was calculated as a measure of the discriminatory power of the model.

All the analyses were done using R v4.1.2 software (R Core Team 2021). The packages glmmTMB and survival were used to fit the models [15,16]. For testing model assumptions, we used the package DHARMa [17]. To test overdispersion, we used the function check_overdispersion of the package performance [18]. Graphs were created using the package ggplot2 [19] and ggsurvfit [20]. Tukey contrasts were performed with the emmeans function of the package emmeans [21].

## 3. Results

### 3.1. Response of Mites to Hypoxia and Humidity

In the first experiment, larvae were exposed to 100 hPa for 10 min. Both species showed a high level of recovery under these conditions. In fact, all larvae recovered, except one larva of *O. attenuata* exposed to air. The second experiment, in which larvae were exposed for different amounts of times to 1 hPa of pressure, showed that time of exposure has a similar influence on survival for both species (Supplementary Table S1). In fact, we found that for each minute of exposure, the probability of survival of an individual larva was reduced on average by 0.21% (CI: 0.04–0.38%).

**Figure 3:**
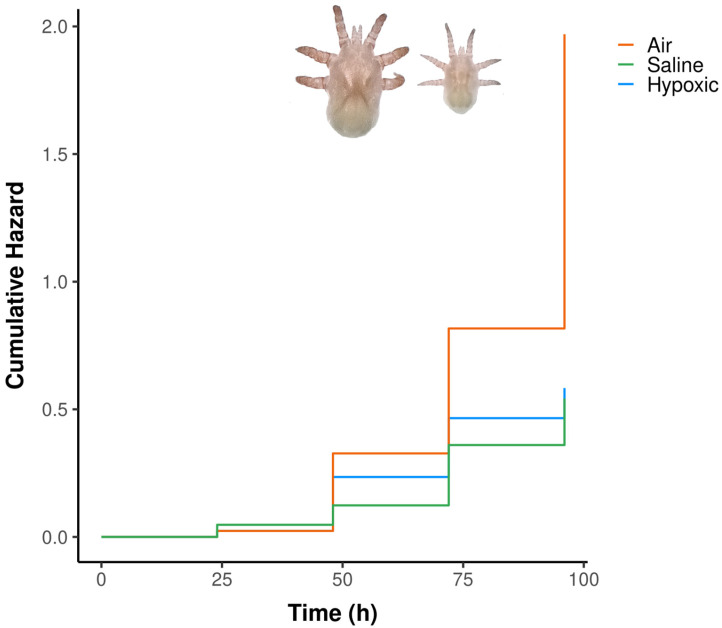
Combined cumulative hazards of *O. attenuata* and *O. diminuata* larvae when exposed to saline solution, hypoxic solution or air.

For the third experiment, in which we evaluated survival using the time for which larvae were exposed to different treatments of hypoxia, we found a high overall significance of the model (Ꭓ^2^_4_ = 22.39, *p* < 0.001). Initially, the air treatment showed a lower risk of dying (99.70% lower risk) compared to the saline solution (β_air_1h_ = −5.90, *p* = 0.077, hazard ratio = 0.003, Supplementary Table S2, Figure 3). However, in contrast with the saline solution, as time progressed, the death hazard for the air treatment increased significantly (β_air_ = 1.64, *p* = 0.0397, hazard ratio = 5.170, Supplementary Table S2, Figure 3). By the end of the experiment, the air treatment showed the highest cumulative hazard. There were no differences between the hypoxic solution and the saline solution (β_hypoxic_sol_ = 0.44, *p* = 0.606, Figure 3), indicating a similar hazard ratio. The concordance of the model was 0.626, indicating a moderately good discriminatory power. Regarding the hypoxic solution, the fact that we opened the vial for every observation did not generate any inflated survival rates, as the proportion of larvae that were alive was similar between the hypoxic solution and the hypoxic control solution at 48 and 96 h (Fischer’s exact test *p* value = 0.6127).

### 3.2. Response of Mites to Salinity

**Figure 4:**
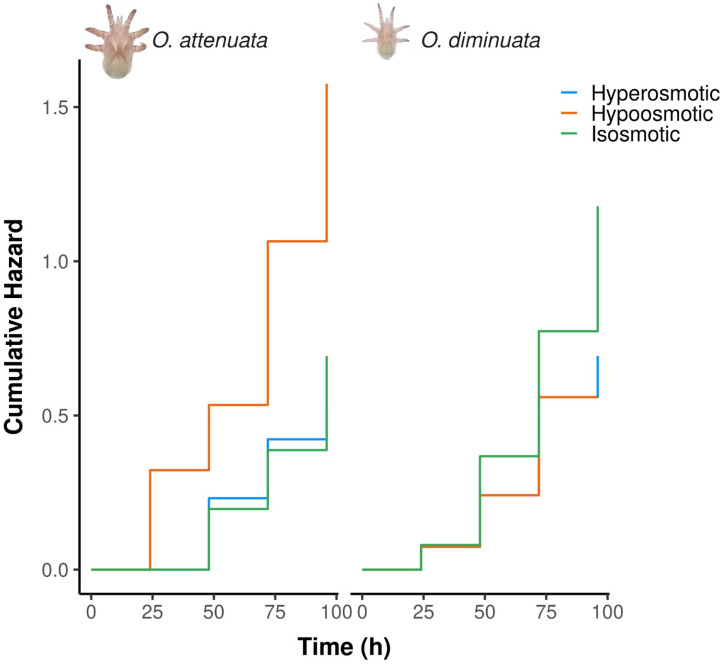
Cumulative hazards of *O. attenuata* and *O. diminuata* larvae when exposed to saline isosmotic solution, hyperosmotic solution or hypoosmotic solution.

The level of salinity in the solution showed that there are differences between species in survival (Supplementary Table S3). For *O. attenuata*, the hyperosmotic solution showed the highest cumulative hazard (Figure 4). In fact, the hazard ratio between the hyperosmotic solution and both the isosmotic and hypoosmotic solutions showed that the hazard rate of *O. attenuata* larvae is higher (Table 3). In contrast, *O. diminuata* showed similar levels of hazard rates between all the treatments and similar levels of cumulative hazard (Table 3, Figure 4). The concordance of the model was 0.627, indicating a moderately good discriminatory power.

## 4. Discussion

In this work, we studied the recovery responses of *O. attenuata* and *O. diminuata* larvae from *A. australis* to simulated host immersion and their survival when exposed to different levels of humidity and salinity. We found that the larvae of both species are very resistant to hypoxia conditions. Also, both species are highly susceptible to dehydration. However, we found that *O. attenuata* is more sensitive to changes in salinity than *O. diminuata*.

We first asked if larvae could survive a short exposure to low oxygen concentration in saline solution in order to understand the effect that apnea during host immersions could have on mite larvae inside nasal cavities. As expected, all larvae except one recovered completely. Then, we exposed larvae to more extreme conditions under increasing exposure times. We found that both species could withstand long times under hypoxia in air (~16 h), although survival decreased over time. Combined, these results indicate that larvae are well adapted to host apnea conditions during immersions. *Arctocephalus australis* requires several immersions to locate food, but their dives usually only last between 6 and 8 min [22]. However, exposure to air for several hours killed some larvae, indicating that humidity is an important factor for survival.

We then studied the long-term survival of larvae exposed to either direct air or hypoxic saline solution in order to understand the influence that humidity has on survival. Our results show that humidity rather than oxygen availability is a constraint for survival. Within the host, larvae are attached to the turbinates’ humid mucosa [4]. The mucosa provides food but also is the medium larvae use in order to take in oxygen. Mite larvae have neither a developed trachea nor a plastron as adults [3]. Although the respiratory mechanism is poorly studied in larvae of these mites, they probably have cuticular respiration [3]. Cuticular respiration in arthropods is common in small, aquatic, or very humid terrestrial species and depends entirely on humidity for oxygen to penetrate the thin, permeable cuticle by diffusion and diffuse into the tissues. If the cuticle dries out, diffusion stops, potentially causing asphyxia. There are several examples of aquatic insects that use cuticular respiration [23]. The fact that larvae survive in hypoxic conditions could indicate that either larvae have a very low metabolism and can survive with just the smallest amount of oxygen that could have entered the vials when we evaluated survival, or that they can survive very long times without exchanging oxygen. Our control series exposing larvae to hypoxic conditions without allowing any oxygen exchange showed results similar to the hypoxic solution treatment, supporting the idea that larvae survive long times without exchanging oxygen, perhaps due to a very low metabolism. In fact, cuticular respiration poses two types of resistance for O_2_ diffusion from water to the tissues. On one side, a water layer just above the respiratory surface, which is deficient in O_2_, provides resistance to diffusion [23]. On the other side, the cuticle itself can act as a diffusion barrier [23]. Hence, arthropods with cuticular respiration are expected to have lower metabolic rates and be smaller in size than arthropods with specific breathing structures [23,24,25].

Parasite mites of semi-aquatic mammals, such as Pinnipeds, must complete their life cycles alternating between terrestrial and aquatic habitats. Mite larvae are at the dispersal phase and are usually expelled when the hosts are ashore during their reproductive or moulting season [26]. Larvae experience a radical environmental change when they transition from being attached to the turbinate mucosa to being outside the host, where they face variable temperature and humidity conditions. Larvae must survive the conditions of the beach long enough to track down a new host. If larvae are expelled on dry sand during the daytime, they will be exposed to direct air and high temperatures. Under these conditions, larvae can survive exposure to high temperatures but must locate a host within the first few days, before low humidity poses a threat to survival ([13] and this work). However, when expelled, larvae may also fall into the intertidal zone, where they will be exposed to conditions very different from those inside the host, such as contact with salty marine water.

Salt water showed to be lethal for *O. attenuata* larvae but not for *O. diminuata*. Our results suggest that both species have different tolerances to osmolality. One possibility is that oxygen diffusion is impaired when exposed to a solution with high osmolality, like salt water. However, our results from the hypoxic solution suggest that both species can tolerate at least 96 h without access to oxygen. Hence, *O. attenuata* seems to be more sensitive to changes in the osmolality of the surrounding solution than *O. diminuata*. Then, detailed descriptions of the cuticles of both species would provide useful information regarding their composition and thickness, and help to better understand what kind of functional protection the cuticle provides for these larvae.

## 5. Conclusions

Our study sheds light on aspects of the ecophysiology of these endoparasites of Pinnipeds that were until now poorly known. Both *O. attenuata* and *O. diminuata* larvae are well adapted to ordinary hypoxia situations, such as when their hosts are diving. In their ability to cope with their host’s foraging behaviour, they show adaptations to the host’s way of life. However, upon being expelled from their host during dispersion, prevailing environmental factors become a threat to their survival. Dehydration and exposure to salt water are two key factors that can limit dispersion, location, and colonization of new hosts.

## Figures and Tables

**Table 1:** Experimental design for testing the effect of hypoxia and dehydration on survival of larvae of *Orthohalarachne attenuata* and *O. diminuata*. N indicates the number of replicates for each species.

Treatment	*O. attenuata* (N)	*O. diminuata* (N)
Saline solution (0.9% NaCl, control)	29	14
Hypoxic saline solution	30	13
Hypoxic saline solution (control)	12	-
Air	30	13

**Table 2:** Experimental design for testing the effect of salinity on survival of larvae of *Orthohalarachne attenuata* and *O. diminuata*. N indicates the number of replicates for each species.

Treatment	*O. attenuata* (N)	*O. diminuata* (N)
Saline solution (0.9% NaCl, control)	29	14
Hypoosmotic solution (~0% NaCl)	28	13
Hyperosmotic solution (3.5% NaCl)	29	14

**Table 3:** Results for *O. attenuata* (upper) and *O. diminuata* (lower) larvae survival in salinity condition experiments. Ratio: Value of the ratio of the contrast. SE: Standard error for the ratio. LCI and UCI: Lower and upper confidence intervals. *p*: *p*-value.

Species	Contrast	Ratio	SE	LCI	UCI	*p*
*O. attenuata*	Isosmotic/Hyperosmotic	0.341	0.122	0.169	0.687	0.003
	Isosmotic/Hypoosmotic	0.832	0.327	0.385	1.799	0.640
	Hyperosmotic/Hypoosmotic	2.440	0.829	1.254	4.750	0.009
*O. diminuata*	Isosmotic/Hyperosmotic	1.183	0.658	0.398	3.520	0.763
	Isosmotic/Hypoosmotic	0.629	0.317	0.234	1.689	0.358
	Hyperosmotic/Hypoosmotic	0.532	0.280	0.189	1.494	0.231

## Data Availability

All data can be found in the Harvard Dataverse repository at https://doi.org/10.7910/DVN/LSLRLV.

## Supplementary Materials

The following supporting information can be downloaded at: https://www.mdpi.com/article/10.3390/biology15050444/s1, Table S1: Analysis of variance table of fitted model for the effect of exposure time on survival of Orthohalarachne attenuata and O. diminuata larvae.; Table S2: Results of fitted model for the survival of Orthohalarachne attenuata and O. diminuata larvae exposed to direct air or hypoxic saline solution.; Table S3: Analysis of variance table of fitted model for the effect of exposure time in solutions with different salinity on survival of Orthohalarachne attenuata and O. diminuata larvae.
