# Supplementary material for: Influence of Hypoxia, Dehydration and Salinity on Survival of Orthohalarachne Marine Mite Larvae: Limits to Dispersion
Source: Biology (Basel). 2026 Mar 9;15(5):444. doi: 10.3390/biology15050444 (PMC12984805; doi:10.3390/biology15050444)
Supplement: Supplementary file 1 [file biology-15-00444-s001.zip › biology-4122594-supplementary.pdf]

Table S1

Analysis of variance table of fitted model for the effect of exposure time on survival of *Orthohalarachne attenuata* and *O. diminuata* larvae. df: degrees of freedom that indicate number of parameters estimated. Chisq: Statistic value. P: p-value, significance at 0.05.

Factor	df	Chisq	P
Species	1	0.1865	0.6659
Time of exposure	1	5.8658	0.0154
Species x Time of exposure	1	0.0103	0.9191

Table S2

Results of fitted model for the survival of *Orthohalarachne attenuata* and *O. diminuata* larvae exposed to direct air or hypoxic saline solution. tt: is the time component of the treatment. Z value: Statistic value. P: p-value. Lower and upper: Lower and upper limit of the confidence interval, significance at 0.05.

Treatment	coefficient	Z value	P	Lower	Upper
Air	-5.897	-1.769	0.077	3.99e-06	1.892
Hypoxic saline solution	-1.921	-0.541	0.589	1.39e-04	154.663
tt (air)	1.642	2.057	0.040*	1.08	24.718
tt (hypoxic saline solution)	0.441	0.516	0.606	0.291	8.311

Table S3

Analysis of variance table of fitted model for the effect of exposure time in solutions with different salinity on survival of *Orthohalarachne attenuata* and *O. diminuata* larvae. df: degrees of freedom that indicate number of parameters estimated. Chisq: Statistic value. P: p-value, significance at 0.05.

Factor	df	Chisq	P
Species	1	0.079	0.7783
Time of exposure	1	5.869	0.0532
Species x Time of exposure	1	6.948	0.0310*
